# The Royal Waterloo Hospital for Women and Children

**Published:** 1903-10-31

**Authors:** 


### THE ROYAL WATERLOO HOSPITAL FOR WOMEN AND CHILDREN.

On Monday, the 26th, the memorial stone of the new buildings was laid by H.R.H. the Duchess of Albany, who was accompanied by Princess Alice and attended by Lady Evelyn Moreing, Mrs. Maxwell, and Colonel Sir Robert Collins. In spite of the inclement weather, a large assembly was gathered together to witness the ceremony. The space available at the hospital on that occasion was so limited that the Board of Governors found, to their regret, that it was impossible to comply with all the requests for tickets. The guard of honour at the hospital was formed by a contingent of the Hon. Artillery Company, lately returned from their trip to America, who were accompanied by their full band and colours. Escorts for the Royal party were drawn from the Surrey Imperial Yeomanry and Middlesex Imperial Yeomanry, whilst the band of the "L" Division Metropolitan Police was stationed inside the building, and played during the time of waiting.

Amongst those on the platform were:—The Lord Mayor and Lady Mayoress, the Sheriffs of London, the Earl and Countess of Denbigh, the Countess of Portsmouth, Lord Denman, the Bishop of Southwark and Lady Barbara Yeatman-Biggs, the Hon. Mrs. Talbot, Viscount Barrington, Lady Llangattock, Lady O'Hagan, Sir Edwin Durning-Lawrence, M.P. (Chairman), and Lady Lawrence, Sir G. Hayter-Chubb, Mr. I. Topham Richardson (Treasurer), and others.

Her Royal Highness was received by the President (the Lord Mayor), Chairman, and Governors of the Hospital, and conducted to the platform on the lower ground-floor, where the first part of the ceremony took place.

The Lord Mayor said that it afforded him great satisfaction to have the privilege, as President of the hospital, of receiving and welcoming her Royal Highness on that auspicious occasion. Her Royal Highness would shortly proceed to lay the memorial stone of the rebuilding of what was the first public institution specially established in the metropolis for the treatment of the diseases of children. At its inception in April, 1816, the institution was located within the precincts of the City of London, and was styled a Dispensary, but it soon outgrew its humble beginnings, and seven years after its foundation, viz., in 1823, his Royal Highness Frederick Duke of York and Albany laid the first stone of the building to be erected on part of the present site. It seemed, therefore, peculiarly fitting and appropriate that her Royal Highness the Duchess of Albany should that day lay the memorial stone of the commodious and extensive hospital which was now being erected to take the place of the smaller building founded in 1823 under the style of the Royal Universal Infirmary for Children.

Before proceeding to lay the memorial stone her Royal Highness would be asked to graciously accept, from some of the ladies and children there present, purses of money which they had been collecting during the last few months, and prior to that he would call upon Sir Edwin Durning-Lawrence, chairman of the committee, to read a short address giving a sketch of the history of the hospital, which they trusted was about to enter upon a course of usefulness upon a greatly extended scale for the benefit of the suffering children and women of that part of the metropolis.

Sir Edwin Durning-Lawrence thanked her Royal Highness on behalf of the committee and governors of the hospital for her gracious presence there that day. Before referring to the history of the hospital he craved permission to express their deep obligation to H.M. the King, who, as Prince of Wales and Duke of Cornwall, consented, for the sake of the suffering women and children there treated, to sell 22 years ago to the trustees of that hospital, the absolute freehold reversion to the site on which the new hospital was now being erected. But for that kind and gracious assent of his Majesty to sell them the reversion of the freehold, a privilege, they believed, never before accorded to anyone upon the estates of the Duchy, they would not have been able satisfactorily to carry on the work of the hospital during the last 20 years, or to undertake its rebuilding on the present extended scale.

He could not omit to mention that his Royal Highness the Duke of Kent (his Majesty's grandfather) presided at the first meeting held in 1816 to establish the original institution. They were very grateful that at all times since its foundation so many members of the Royal Family had taken the deepest interest in its welfare.

They were met there that day, not to speak of the past value of the hospital, which had been very great, but to inaugurate the commencement of a new epoch of vastly extended usefulness, and he trusted that her Royal Highness might be spared many years to look back with satisfaction and pleasure to the part she had so graciously played that day in placing in the front pier of the building a fitting memorial stone to commemorate the foundation of the new Royal Waterloo Hospital for Women and Children.

Mr. I. Topham Richardson (treasurer) then drew attention to the present financial position of the hospital, and referring to the building fund stated that a balance of £32,764 was still required towards the total amount of £50,000 wanted for the new hospital. Having thanked the Duchess of Albany for the kind and valuable interest she had exhibited in that institution for many years past, culminating in the honour which her presence there that day had done them, he wished to testify to the distinct success which had so far attended the zeal and ability displayed by the Ladies' Committee, of which her Royal Highness was graciously acting as Chairman, with the Countess of Derby as Chairman of the Finance Committee, Mrs. Henry Tate, Hon. Sec.; Miss Lewis, Assistant Hon. Sec.; and Sir George Hayter-Chubb, Treasurer. They had been greatly encouraged by this result in their efforts to obtain the sum which was still required for the completion of the entire building.

Dr. W. J. Gow expressed, on behalf of the medical staff, their thanks to her Royal Highness for so graciously consenting to lay the foundation stone of the new building. The need for increased hospital accommodation in that part of London was recognised by all, but perhaps by none so keenly as by those who were brought into contact with the sick poor. This was a hospital not only for children, but for women also, and in both departments they might claim to do useful and creditable work, and that they supplied a real want was evidenced by the large and increasing number of patients who sought relief there, drawn not only from the south of London, but from many of the adjacent counties. The resources of the hospital had in the past been greatly strained to meet the urgent demand for in-patient accommodation, as well as to deal with the vast number of out-patients who came for treatment. They hoped in the new building not only to be able to deal with a larger number of patients, but also to deal with them in a manner more consistent with modern medical and surgical methods. Light and air were as essential to the sick as to the well. They hoped and believed that in the new building they would be able to extend the efficiency of the institution, and that when it was completed it would be second to none in the kingdom in all the essentials which were conducive to the well-being of the patients.

A good number of ladies and children then presented purses to the Duchess, and Sir George Hayter-Chubb announced that the total sum thus obtained was £2,500.

Sir Edwin Durning-Lawrence, on behalf of her Royal Highness, stated how much pleasure it had given her to receive the purses from the dear children, and how much obliged she felt to the ladies who had aided in collecting so large a sum for the hospital, and trusted they would not forget all they owed to Mrs. Tate and Miss Lewis for the splendid work they had done as hon. secretaries.

The whole assembly then adjourned to the upper ground-floor where a photograph was taken of the gathering. The Lord Mayor having presented her Royal Highness with a handsome silver trowel, the Duchess of Albany laid the memorial stone, and affirmed in a clear distinct voice "I declare this memorial stone to be well and truly laid." Prayers were then offered by the Bishop of Southwark.

A vote of thanks to her Royal Highness was proposed by the Lord Mayor and seconded by Sir George Hayter-Chubb, after which hearty cheers were given. The Royal party then left the hospital, having first partaken of tea.

The memorial stone, which was of polished Aberdeen granite, and was let into the Waterloo Bridge Road front of the building on the ground-floor, bore the following inscription:—"This stone was laid by H.R.H. Helen Duchess of Albany, October 26th, 1903." Thereafter followed a list of the names of the President, Chairman, Treasurer, and members of the committee. Beneath the stone was buried all coins of this year up to £1, supplied by Parr's Bank, and a copy of the history of the hospital.

The attention of the visitors was called to an incrusted glass tablet showing date of foundation, which was discovered when excavating for the foundations of the new hospital.

